# Adolescent wellbeing after COVID-related school-closings

**DOI:** 10.3389/fpsyg.2026.1591897

**Published:** 2026-03-27

**Authors:** Lars Michael Göllner, Jessica Rademacher, Simon Forstmeier, Ina Fassbender

**Affiliations:** Department of Psychology, University of Siegen, Siegen, Germany

**Keywords:** adolescence, COVID-19, life-satisfaction, lockdown, loneliness, resources, self-efficacy, social support

## Abstract

**Introduction:**

Research has shown that the COVID-19 pandemic and related lockdowns led to increased loneliness and decreased life-satisfaction among adolescents. However, the impact was not uniform, suggesting that certain resources may have served as protective factors. This study adopts a resource-based perspective to identify predictors of adolescent well-being after and at the end of the second COVID-related lockdown, focusing on self-efficacy, social support, and religiosity, as well as the role of a disposition to help.

**Methods:**

Using data from a longitudinal sample of 171 German-speaking adolescents aged 15–18 y., assessed at four time points over 6 months, we analyzed which resources during the lockdown period were associated with life-satisfaction and loneliness at the final assessment.

**Results:**

Our findings indicate that self-efficacy and general social support were significant predictors of higher life-satisfaction, while peer support uniquely predicted reduced loneliness. Parental support also positively influenced life satisfaction when general social support was excluded from the analyses.

**Discussion:**

The findings underscore the crucial role of peers and family as primary sources of support during the pandemic, even in contexts of physical distancing. This study highlights the need to differentiate between types of social support and distinct aspects of well-being, such as life-satisfaction and loneliness. It underscores self-efficacy and peer support as central resources for fostering resilience in adolescents during crises. These insights provide valuable guidance for developing targeted prevention and intervention programs to strengthen adolescents’ resources and support systems in preparation for future disruptions or lockdown scenarios.

## Introduction

Since the outbreak of the COVID-19 pandemic, numerous international studies have explored its impact on various aspects of life, including physical health, the economy, politics, and social equality. Mental health garnered significant attention due to COVID-related challenges, global lockdowns, and associated (social) isolation. Studies found increases in mental health issues, especially in the initial stage of the pandemic, but also in psychological problems, fear, loneliness, and social isolation, and a reduction of life-satisfaction in different parts of the population shortly after the pandemic outbreak (Arora et al., 2020; Buecker and Horstmann, 2021; Clair et al., 2021; Ernst et al., 2022; Hossain et al., 2020; Paczkowska et al., 2022; Pai and Vella, 2021; Robinson et al., 2022; Talevi et al., 2020). However, some individuals demonstrated notable resilience, with loneliness levels declining faster than anticipated (Luchetti et al., 2020).

The desire for autonomy, independence from parents, and the need for social relationships and peer experiences are vital components of the daily routines, mental health, and life-satisfaction of children and adolescents. These developmental needs were significantly disrupted by COVID-related lockdowns and distance learning (Bülow et al., 2021; Gadassi Polack et al., 2021). As a result, children, adolescents, and young adults were particularly vulnerable to the negative effects of lockdowns and school- or college-closings, which manifested in increased depression, anxiety, distress, and loneliness (Bu et al., 2020; Haikalis et al., 2022; Viner et al., 2022; Werling et al., 2022). Studies consistently found that identifying as female and being younger were among the strongest predictors of reduced wellbeing during the pandemic, emphasizing the disproportionate psychological toll on these groups.

While several studies have highlighted the negative psychological effects of lockdowns and school closings on adolescents — including declines in life satisfaction and increases in internalizing symptoms — emerging evidence also points to notable resilience in this age group. For instance, longitudinal studies observed that young people’s wellbeing returned to pre-pandemic levels by the summer of 2020 (Breaux et al., 2021) and some reported stable or even improved indicators of mental health during lockdowns compared to pre-pandemic baselines (Chaffee et al., 2024; Kaman et al., 2024). In population-based data from Germany, the longitudinal COPSY study (Kaman et al., 2025) documents a clear deterioration in mental health and quality of life among children and adolescents during the COVID-19 pandemic compared to pre-pandemic reference data, and – despite some recovery in later waves – the prevalence of psychological burden remains elevated years after the onset of the pandemic. This evidence of overall decline combined with findings of individual resilience highlights the need to better understand which individual and contextual factors supported positive adjustment during this period – such as those examined in the present study.

### Protective factors against mental health issues during COVID-19 pandemic

The literature highlights both risk and protective factors influencing individual wellbeing during the pandemic. Beyond demographic risk factors (e.g., age, gender) and premorbid health factors (e.g., physical health), key protective factors such as self-efficacy, social support, and religiosity have been identified as significant contributors to maintaining mental health during crises (Deighton et al., 2019; Hysing et al., 2007). However, research specifically focused on adolescents’ protective resources during COVID-19 lockdowns remains limited. This study aims to address this gap by investigating which factors helped protected adolescents’ wellbeing during these challenging circumstances.

Self-efficacy, defined as an individual’s belief in their ability to influence their life or cope with challenges, plays a critical role in promoting wellbeing and life-satisfaction. Bandura & Watts (1996, p. 1) emphasize that a lack of control over adverse circumstances can lead to anxiety, apathy, or despair. Self-efficacy is associated with better mental health outcomes and fewer pathological symptoms (Dupéré et al., 2012; Najafi and Foladjang, 2007). In the context of the COVID-19 pandemic, self-efficacy emerged as a crucial protective factor for young adults. Hussong et al. (2021) demonstrated in a longitudinal study that high self-efficacy buffered against increases in mental health symptoms, including internalizing and externalizing problem behaviors, during the early months of the pandemic in the United States. Similarly, Yıldırım and Güler (2022) found that self-efficacy predicted mental health outcomes above and beyond factors such as age, gender, and chronic illnesses. Before and during the COVID-19 pandemic, low self-efficacy has been associated with greater loneliness and lower life-satisfaction (Azizli et al., 2015; Çakar, 2012; Roth et al., 2021; Suanet and van Tilburg, 2019). These findings underscore the critical role of fostering self-efficacy, particularly in challenging times, to support mental health and wellbeing.

Religiosity, often considered a significant resource for mental health and life-satisfaction, encompasses multiple dimensions, including beliefs about transcendence, the transcendent meaning of life, and participation in religious practices (Erci and Aktürk, 2018). Numerous studies highlight the positive impact of religious commitment on mental health, particularly in preventing, coping with, and recovering from mental or physical illnesses (Gartner et al., 1991; Matthews et al., 1998). In the context of COVID-19, religiosity has shown specific benefits. It has been associated with better coping mechanisms for pandemic-related stress (Agbaria and Abu-Mokh, 2022) and greater psychological resilience, reducing fear of COVID-19 (Batmaz and Meral, 2022). Religious belief systems offer existential answers, meaning, and purpose, which can be especially helpful during crises. Additionally, they often provide a sense of community and social support (Moxey et al., 2011). These findings underline the multifaceted ways in which religiosity contributes to mental wellbeing.

Social support serves as a critical coping resource during stressful events or situations. A meta-analysis by Rueger et al. (2016) highlighted that perceived social support from diverse sources - family, teachers, and peers - is connected to lower levels of depression in children and adolescents. Importantly, these sources of support appear to function independently, collectively reducing emotional problems caused by loneliness, social isolation, and suicidal thoughts in adolescents (Helsen et al., 2000). The COVID-19 pandemic offered a unique context for examining social support networks. With self-isolation becoming necessary, seeking and offering support became more challenging as physical meetings posed potential risks. This shift contributed to increased feelings of loneliness globally, particularly among adolescents and young adults (O’Sullivan et al., 2021; Sabato et al., 2021). However, individuals who experienced strong support from family and friends reported fewer mental health issues and lower perceived stress during lockdowns (Agbaria and Abu-Mokh, 2022; Panteli et al., 2021; Qi et al., 2020; Szkody et al., 2021). Especially (virtual) face-to-face interactions emerged as a protective factor against loneliness, demonstrating that even during periods of physical separation, social connections remain crucial (Sabato et al., 2021).

Only few studies have been conducted on the (virtual) practical, learning, and psychosocial support provided by teachers and schools. Hatzichristou et al. (2021) revealed that students with inadequate technological resources at home reported a lack of school support during distance learning. While some students felt supported by their schools, others experienced significant gaps in assistance. Close virtual teacher-student relationships, marked by warmth, trust, and emotional support, proved to be a protective factor against mental health challenges. For instance, Ye et al. (2022) demonstrated that students with strong teacher-student relationships were less affected by cyberbullying in terms of mental health difficulties compared to peers with weaker relationships. Additionally, teacher support during distance learning also promoted academic resilience, enabling students to succeed academically despite the challenges of remote education (Permatasari et al., 2021). These findings underline the importance of teacher support as a resource in overcoming educational and emotional problems during crises, prompting further investigation into its protective role during the COVID-related lockdowns.

Although the present study focusses on the context of the COVID-19 pandemic, the protective role of self-efficacy and social support for adolescent wellbeing is well established across diverse non-pandemic settings. Theoretical frameworks such as self-determination theory emphasize the central importance of competence and relatedness for psychological adjustment (Deci and Ryan, 2000), and resilience research has consistently identified supportive relationships and personal efficacy beliefs as core protective factors in the face of adversity (Masten et al., 2012). Moreover, associations between social support and loneliness, as well as the developmental salience of peer relationships during adolescence, have been widely documented (Hawkley and Cacioppo, 2010; Rueger et al., 2016). Against this background, the present study not only examines these resources during a period of societal disruption but also provides an opportunity to explore whether established developmental processes continue to operate under conditions of prolonged stress and structural restriction.

### Adolescents’ disposition to help during COVID-19 pandemic

Governments worldwide implemented measures such as vaccination, social distancing, and mask-wearing to reduce the spread of COVID-19. Studies found that individuals with higher levels of empathy and prosocial behavior were more likely to adhere to these protective measures (Jordan et al., 2021; Pfattheicher et al., 2020). Prosociality refers to voluntary actions aimed at benefitting others (e.g., caring, donating, helping, sharing), while empathy involves recognizing and responding to others’ emotions and needs (Fassbender and Luhmann, 2021; Paulus, 2009). Empathy not only facilitated compliance but also fostered optimism during the pandemic (Franke and Elliott, 2021). Prosocial behaviors were linked to higher positive affect early in the pandemic (Varma et al., 2022). It is hypothesized that empathic and prosocial individuals identified more strongly with the public health measures, viewing them as personal contributions to combating the pandemic. Among college students, strict adherence, particularly social distancing, increased feelings of loneliness (Vaterlaus, 2022). Other studies confirmed elevated loneliness levels in adolescents and young adults - likely due to the social isolation necessitated by the measures (Bu et al., 2020; Groarke et al., 2020). Further, Qu et al. (2022) demonstrated that empathic adolescents engaged more actively in preventive health behaviors, such as wearing masks and maintaining social distance, but they also experienced higher levels of COVID-19-related preoccupation. These mixed findings highlight the complexity of empathy and prosociality in the context of pandemic-related compliance and mental health outcomes. Due to the divergent results of previous studies, the present study investigates whether a disposition to help, encompassing empathic and prosocial behaviors, predicts life-satisfaction and loneliness after the second COVID-related lockdown with school-closings.

### The present study

**Figure 1:**
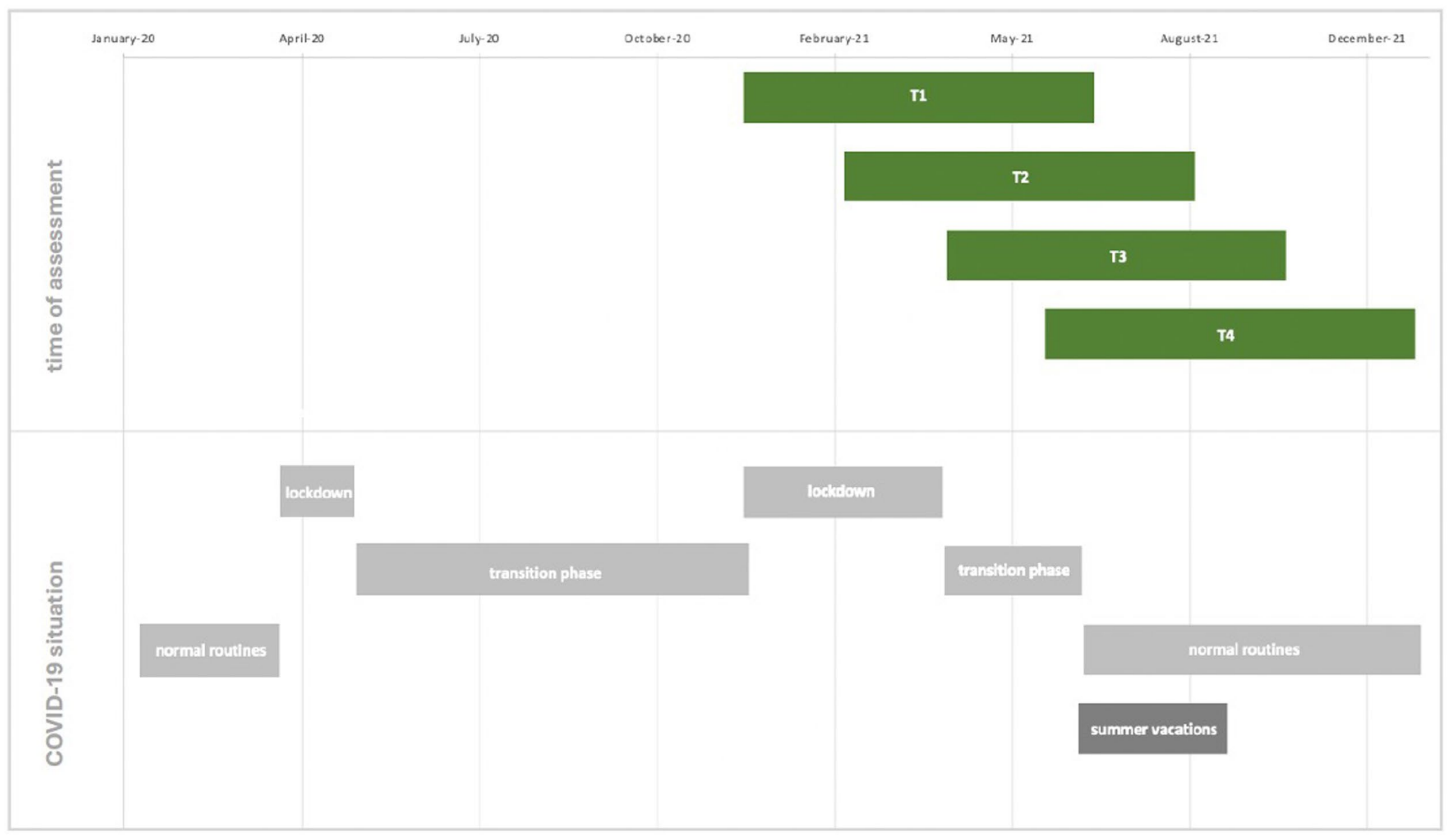
Gantt chart of the four assessments and corresponding COVID-19 situation phases.

A resource-based perspective on adolescents’ experiences during the COVID-19 pandemic, particularly following the second lockdown with school-closings and during the subsequent transition phase, offers critical insights into protective factors for life-satisfaction and against loneliness. The objective of this study is to identify potential starting points for preventive interventions aimed at supporting adolescents during future lockdowns or other critical life events. This study utilizes data from the longitudinal [YouChanged?!]-Study, comprising 171 German-speaking adolescents aged 15–18 who participated in all four assessments conducted over a six-month period, with data collected every 8 weeks (T1–T4). This broader longitudinal study was designed to address multiple research questions, including change processes across repeated assessments. While several constructs were measured across three or four waves to enable longitudinal analyses in related projects, the present manuscript focuses on prospective associations between earlier resources and wellbeing at T4. This design allows temporal separation between predictors and outcomes, thereby reducing common method bias. The study started on December 15, 2020, coinciding with the onset of the second COVID-19 lockdown in Germany, and closed for new participants in June 2021, when schools began returning to in-person classes. Consequently, the final T4 assessments were completed in December 2021. The T1 assessment was conducted during the lockdown or the early transition phase, while T4 occurred at the end of this phase or after the resumption of normal routines post-lockdown. During this transition phase, school reopening strategies were formally determined by the federal states, but the actual implementation varied widely across school districts and individual schools, depending on local infection rates and administrative decision (Sonnenburg et al., 2022). However, due to data protection, participants’ specific locations were not recorded, precluding precise identification of their school reopening timelines. Depending on when participants joined the study, T2 and T3 assessments could have taken place during lockdown, the transition phase, or the post-lockdown period - including the transition period, post-lockdown school reopening, or summer vacation, which last 6 weeks in Germany and start at different timepoints in each federal state (see Figure 1 for a detailed timeline of assessments and the corresponding COVID-19 context; see Sonnenburg et al., 2022, for more details on the structure and variation of school regulations during the pandemic). Consequently, in the context of this study, the term “transition phase” refers to the period following the second strict nationwide lockdown in Germany (beginning in early 2021), during which schools gradually reopened under heterogeneous regulations that varied not only between federal states but also across school districts and, in some cases, individual schools. This phase was characterized by a complex mixture of in-person, hybrid, and remote teaching formats, as well as alternating attendance models and evolving hygiene regulations. In our analysis, we investigate resources assessed at T1, T2, and T3, that are associated with levels of loneliness and life-satisfaction at T4, i.e., at the end of the transition phase and after the second lockdown with school-closings (Research Questions 1 and 2). Additionally, we explore the relationships between adolescents’ disposition to help and their life-satisfaction, as well as between their disposition to help and loneliness (Research Questions 3A and 3B; see Table 1 for a summary of the assessments).

**Table 1:** Assessment of predictor and outcome variables used in this study.

Type	Variable	T1	T2	T3	T4
Predictors	Religious belief	X			
	Self-efficacy			X	
	General social support		X		
	Teacher support		X		
	Parental support			X	
	Classroom support			X	
	Peer support			X	
	Prosociality				X
	Perspective taking				X
Outcomes	Life-satisfaction				X
	Loneliness				X

Overview of the different times of assessment (T) of the predictor and outcome variables.

### Research questions and hypotheses

Based on previous research, we formulated hypotheses regarding the associations between the resources—self-efficacy, religious belief, and social support – and adolescent wellbeing, operationalized as life-satisfaction and loneliness. Additionally, we explore the potential relationship between a disposition to help (prosociality and empathy) and both loneliness and life-satisfaction.

#### Hypotheses

The first objective of this study is to determine whether adolescents with stronger resources experienced higher life-satisfaction (Research Question 1) and lower loneliness (Research Question 2) after the second COVID-related lockdown with school-closings and at the end of the transition phase, compared to peers with fewer resources. Previous studies indicate a protective role of various resources in buffering against diminished wellbeing. For the purposes of this study, we conceptualize resources as perceived social support, self-efficacy, and religious belief during the lockdown and transition phases. Specifically, we hypothesize that higher levels of religious belief (measured at T1), perceived social support (measured at T2 or T3), and self-efficacy (measured at T3) will predict greater life-satisfaction at T4 (Hypothesis 1) and lower loneliness at T4 (Hypothesis 2).

#### Exploratory research question

The second objective is to investigate the role of empathy and prosociality – dimensions of a disposition to help – in predicting loneliness and life-satisfaction, with all variables assessed at T4. Previous studies have reported mixed findings on the effects of prosocial tendencies and empathy on loneliness and life-satisfaction in the context of COVID-related lockdowns (Cho et al., 2022; Espinosa et al., 2022; Qu et al., 2022). We aim to clarify these relationships by exploring whether a disposition to help predicts loneliness (Research Question 3A) and life-satisfaction (Research Question 3B).

## Methods

Our sample was taken from the four-wave longitudinal [YouChanged?!]-Study on life event perception, wellbeing, and personality development in adolescence conducted by two of the authors at University of Siegen. The study design had been preregistered on December 15, 2020^1^. At the moment of preregistration, we had never worked with the data used in this study. Before manuscript submission, partial findings of this study have been presented at a conference. The [YouChanged?!]-Study data set was created on January 11, 2022. We preregistered the present analyses at https://osf.io/e2dgc/registrations. Certain sections of the manuscript were revised using ChatGPT (Version 2024–12), an AI-based writing assistant developed by OpenAI. Its use was limited to stylistic and grammatical refinements; the scientific content and interpretations were not affected. All suggestions generated by the AI were manually reviewed and integrated into the overall context.

### Study design and data collection

The adolescents were recruited for an age range of 15 to 18 years allowing participations for peers +/−1 year of age. Minors (under 18 years of age) needed online parental consent to participate in both the study and a voucher raffle; adolescents of age provided online consent themselves. The study consisted of four assessments within 6 months, i.e., every 8 weeks (T2 was 8 weeks, T3 was 16 weeks, and T4 was 24 weeks after T1). For each assessment, the participants automatically received an invitation-email with an individual link to their next assessment. If the link had not been opened within a week, they received a reminder e-mail allowing for another answer frame of 1 week. The questionnaire had to be answered in one go, but there was no time limit. However, in data cleaning, we excluded participants with very short survey times, i.e., less than 4 min, in view of an average survey duration of 8.9 min, at every assessment to ensure data quality. Participants could leave out single assessments without being dropped from the study. To motivate participation, there was a raffle of 10 20€ vouchers and 1 50€ voucher after each assessment and in a final raffle of 5,100€ vouchers for which each participation at T1-T4 provided a raffle ticket. The questionnaires were implemented in SoSciSurvey (Leiner, 2019). As data collection started right when the second lockdown against COVID-19 started and schools shifted to distance-learning, all participants started the study in a situation of social distancing. German-speaking adolescents were recruited exclusively online through personal contacts, flyers, social media, local newspapers, and parent councils of German secondary schools. The adolescents needed access to a device with a stable internet connection to participate. When schools started to return to in-person classes in June 2021, recruitment was stopped to ensure that for all participants, T1 had been during lockdown or in the transition phase from lockdown to normal routines. Consequently, the last T4-questionnaire was answered on December 22, 2021, and the study was closed shortly after. As noted above, assessments at T2 and T3 may have taken place during different pandemic-related contexts, including periods of lockdown, transition phase, or school holidays. This temporal heterogeneity may have influenced adolescents’ perceived social support and self-efficacy, as the availability and relevance of specific support sources likely varied across these contexts. Consequently, results related to these predictors should be interpreted with caution. The dependent variables were collected at T4, i.e., at the end of the transition phase and after the second COVID-related lockdown with school-closings. We included participants with scale scores on at least life-satisfaction or loneliness, ideally both, at T4.

### Participants

During data cleaning (initial sample size of *N* = 370 at T1), we excluded participants based on following criteria: (a) participants who provided at least one false answer on instructed response items at T1-T4 (*n* = 61), (b) participants who dropped out of the study before T4 or did not provide data on at least one of the two outcome variables at T4 (life-satisfaction or loneliness; *n* = 105,), (c) participants with very short survey durations, i.e., less than 4 min (*n* = 3) at any assessment, (d) participants exceeding the designated age range (younger than 14 or older than 19; *n* = 27), and (e) participants identifying as “diverse” on the gender item (i.e., the third out of three gender categories in the German civil registry) (*n* = 3), as this group was too small to allow meaningful statistical analysis. Participants without T4 outcome data were excluded using listwise drop-out, in line with our preregistered analysis plan. Since life-satisfaction and loneliness were assessed only at T4 and not at earlier timepoints, imputing outcome data was not deemed appropriate. Although attrition reduced the final sample size and may introduce biases if dropout was for any systematic reason, it remained sufficient to detect medium-sized effects with adequate power (see Power Analysis section). Participants without the critical scale scores at T4, e.g., for leaving the study prior to T4, did not differ significantly in general social support [*t*(63.54) = −0.83 *p* = 0.41] teacher support [*t*(51.85) =–0.29 *p* = 0.77], parental support [*t*(27.91) = 0.31 *p* = 0.76], peer support [*t*(30.45) = 0.07 *p* = 0.95], classroom support [*t*(28.60) = 0.31 *p* = 0.76] or self-efficacy [*t*(33.00) = 0.52 *p* = 0.61] from the participants of the final sample providing these scale scores. We may thus assume that they did not drop out for any systematic reason.

The final sample consisted of *N* = 171 participants between 14 and 18 years of age (*M* = 16.54; *SD* = 1.19; 76.4% female, 23.6% male). Participants were asked to freely name their religion in a non-mandatory open-answer field. 76.27% could be identified as Christian, 19.5% as Atheist, and 1.7% as Muslim. 2.5% did not provide any (valid) information. We conducted an *a priori* power analysis using G*Power Version 3.1.9.6 (Faul et al., 2007). We planned the analyses outlined below with the anticipation that, with a maximum sample size of *N* = 171 at T4, we would be able to detect a medium effect size with 80% power while using up to nine predictors, necessitating a strict significance level of *p* < 0.01. The a priori power analysis indicated that a regression analysis with nine predictors would require a sample size of *N* = 154. As the final sample size exceeded this minimum requirement, statistical power was sufficient. For completeness, we additionally conducted *post hoc* power analyses to confirm the adequacy of power for the reported models, especially given the reduced sample size after data cleaning. The results indicated power levels of 0.99 for the main analyses utilizing the *R*-package *pwr* (Champely et al., 2017).

### Measures

#### Predictors

For all scales listed subsequently, we calculated scales means, with higher scores indicating higher levels of the assessed predictor values. We used the R-package *psych* (Revelle, 2022) to calculate internal consistencies.

##### Religious belief

Religious belief was assessed with the first scale of the *System of Belief Inventory* (SBI) by Albani et al. (2002) at T1 (*n* = 68). The participants answered 10 items on a 4-point Likert scale ranging from 1 (agree strongly– trifft vollständig zu) to 4 (disagree strongly – trifft gar nicht zu) (e.g., ‘I have experienced a sense of hope as a result of my religious or spiritual beliefs‘). The scale showed a good internal consistency (*α* = 0.86).

##### Social support

General social support was measured with a self-developed shortform of the *Social Support Questionnaire* (F-SozU) by Fydrich et al. (2009) at T2 (*n* = 151 participants answered all items). We only used 8 out of 14 items (e.g., ‘I experience a lot of understanding and security from others‘) because 6 items were not applicable in the lockdown situation. They referred to meeting or hugging friends^2^. The items were answered on a 5-point Likert scale from 1 (not true – trifft nicht zu) to 5 (exactly the truth – trifft genau zu) and assessed perceived support from the social environment. The F-SozU items had a good internal consistency (*α* = 0.89), calculated based on the eight items included in the final analyses.

Teacher support was measured with a subscale of the *Student Social Support Scale* (SSSS) by Malecki and Elliott (1999) at T2. *n* = 128 participants answered all 6 items (e.g., ‘My teacher explains things when I’m confused.’). The 6-point Likert scale ranging from 1 (never – niemals) to 6 (always – immer). Since there is no German version of the scale and given the lack of validation, we chose the items according to content criteria and translated the items using the back-translation method (Brislin, 1970). The SSSS showed a good internal consistency (*α* = 0.87).

Parental support (*n* = 155 participants answered all items), classroom support (*n* = 154 participants answered all items), and peer support (*n* = 155 participants answered all items) were assessed with the respective subscales of the *Questionnaire on resources in childhood and adolescence* by Lohaus and Nussbeck (2016, FRKJ 8-16) at T3. For classroom support (e.g., ‘My classmates are kind to me‘) and peer support (e.g., ‘I have friends I can rely on‘), all six items were used. For parental support (e.g., ‘Whenever I need support, my parents are there for me‘), only the three items with the strongest item discrimination (out of six) in the original publication were used to ensure the most efficient processing time of the questionnaire. All items could be answered on a 4-point Likert scale ranging from 1 (never agree – stimmt nie) to 4 (always agree – stimmt immer). The subscales showed satisfying to good internal consistencies (parental support α = 0.87, classroom support α = 0.85, peer support α = 0.74).

##### Self-efficacy

A 6-item subscale of the FRKJ 8–16 (2016) assessed self-efficacy at T3 (*n* = 155 participants answered all items) on a 4-point Likert scale ranging from 1 (never agree – stimmt nie) to 4 (always agree – stimmt immer) An example item is ‘I can achieve everything with my abilities‘. The internal consistency was very good (*α* = 0.94).

#### Outcomes

##### Life-satisfaction

The participants rated their life-satisfaction on the *Students‘Life-satisfaction Scale* (SLSS), by Huebner (1991; Weber et al., 2013) at T4 (*n* = 169 participants answered all items). The scale consists of 7 items (e.g., ‘My life is going well‘) rated on a 6-point Likert scale ranging from 1 (strongly disagree – stimme überhaupt nicht zu) to 6 (strongly agree – stimme stark zu). The scale showed a very good internal consistency (α = 0.91).

##### Loneliness

We used the 9-item version of the *UCLA Loneliness Scale* (Luhmann et al., 2016) to assess the individual’s subjective feelings of loneliness at T4 (*n* = 169 participants answered all items). Nine items (e.g., ‘How often do you feel left out?’) were answered on a 4-point Likert scale ranging from 1 (never – niemals) to 4 (always – immer). The internal consistency of the scale was good (α = 0.86).

#### Further constructs for exploratory analyses

##### Disposition to help

We defined the construct of disposition to help as an index combined by scales assessing prosociality and perspective taking. In the [YouChanged?!]-Study, the 6-item short form of the *German Prosociality Scale* by Fassbender and Luhmann (2021) was used. The short scale consists of two facets: active-helping behavior and emotional-empathetic behavior with 3 items each. The items (e.g., ‘I try to help others‘) could be answered on a 5-point Likert scale ranging from 1 (never - nie) to 5 (always - immer) at T4 (*n* = 170 participants answered all items). Perspective taking was assessed with a short form of the subscale Perspective Taking of the German version of the *Interpersonal Reactivity Index* by Paulus (2009) also used in Fassbender and Luhmann (2021). The short form consists of 3 items (e.g., ‘I sometimes find it difficult to see things from the other person’s point of view.‘) that were answered on a 5-point Likert scale ranging from 1 (never - nie) to 5 (always - immer) at T4 (*n* = 173 participants answered all items). The internal consistencies of the scales were acceptable (α = 0.54–0.68), probably because the mathematics behind Cronbach’s Alpha significantly makes higher coefficients less likely for short scales (Ziegler et al., 2014). We conducted two confirmatory factor analyses^3^ to test whether the two facets of prosociality and perspective taking with three items each loaded on one factor, representing a disposition to help as one construct, or on separate factors. To test the model fit we used the χ^2^ fit statistics and the criteria outlined by Schermelleh-Engel et al. (2003): TLI ≥ 0.95, RMSEA ≤ 0.08, and SRMR ≤ 0.10, and only factor loadings ≥ 0.4 were interpreted. We did not find a unidimensional disposition to help (χ^2^_27_ = 95.32, TLI = 0.643, RMSEA = 0.123, SRMR = 0.087). The second confirmatory factor analysis with the three scales as separate factors comprising 3 items each yielded good fit (χ^2^_24_ = 38.00, TLI = 0.92, RMSEA = 0.06, SRMR = 0.046). Consequently active-helping behavior, emotional-empathetic behavior, and perspective taking need to be treated as separate scales. A detailed description of the factor analyses with path diagrams can be found in the Supplemental material (c.f. *Preliminary Analyses* and Supplementary Figures S1, S2).

### Analyses

We used the R-packages *car* (Fox and Weisberg, 2022), *dplyr* (Wickham et al., 2022), *psych* (Revelle, 2022), and *readxl* (Wickham and Bryan, 2022) for data preparation and to calculate scale scores. Due to listwise dropout, only data of participants who had answered all relevant items at the respective assessment were included. Supplementary Table S2 lists the final sample sizes and assessment points for all scales. Regarding the SBI, it is important to note that the items had not been displayed to all participants. There was a filter question asking participants to rate their religiosity on a scale from 1 (not at all – gar nicht) to 5 (very much – sehr). Only those who had selected 3 or higher were displayed the religious belief inventory and could have obtained a scale mean when answering all items. Therefore, there are only *n* = 68 participants in the analyses containing the SBI.

Previous studies found gender effects in prosociality, empathy, perceived teacher support, and COVID-related suffering (Guadagni et al., 2020; Silke et al., 2018). Female participants showed more COVID-related anxiety and depression, empathy, prosociality, and they perceived more teacher support. Regarding age, there is uncertainty in the decrease of children’s and adolescents’ wellbeing during the COVID-related lockdowns (Newlove-Delgado et al., 2021). Because of possible gender and age effects in the constructs of interest, we tested the dependent variables for main effects of gender and age as preliminary analyses. In our main analyses, we inspected the correlation of the eight predictors (self-efficacy, religious belief, five forms of perceived support, and gender) and the two outcomes (life-satisfaction, loneliness). We calculated multiple regression models for Hypothesis 1 and 2. Regression tables were printed using R-package *apaTables* (Stanley, 2021). As preregistered, we explored Research Questions 3A and 3B inspecting scale correlations. Only in the case of significant correlations, we would conduct a multiple regression model for the respective question. Type I error probability was reduced by using a conservative *p* < 0.01 criterion for statistical significance in the multiple regression models, given the high number of predictors (Groarke et al., 2020). For *t*-tests, correlation analyses, and our exploratory analyses, we used the standard *p* < 0.05 criterion to determine statistical significance (Al Omari et al., 2021).

## Results

### Descriptive statistics

Using the R-packages *car* (Fox and Weisberg, 2022), *lmTest* (Zeileis and Hothorn, 2002), and *olsrr* (Hebbali, 2020), we tested for linearity, correlations between the residuals, multicollinearity, homoscedasticity, and normal distribution prior to conducting multiple regression models.

### Preliminary analyses

When testing for main effects of gender and age, loneliness (*β* = 0.16, *p* = 0.038) and life-satisfaction (*β* = −0.18, *p*  = 0.021) were significantly predicted by gender. Male gender predicted more life-satisfaction while female gender predicted more loneliness. There were no main effects for age in life-satisfaction (*β* = −0.03, *p* = 0.692) and loneliness (*β* = 0.12, *p* = 0.133). Hence, as preregistered, we did not include age as a covariate in the main analyses, but gender only.

### Main analyses

We calculated Pearson-correlation coefficients for the bivariate relations of predictors and outcomes (displayed in Table 2). There were significant correlations for all predictors and life-satisfaction except for religious belief. The same pattern emerged for the predictors correlated with loneliness. Consequently, religious belief was not included in the subsequent regression analyses. To test Hypotheses 1 and 2, we calculated multiple regression models with the predictors general social support, teacher support, parental support, classroom support, peer support, self-efficacy, and gender for both outcomes life-satisfaction and loneliness (cf. Tables 3, 4). We had hypothesized a higher religious belief at T1, a higher perceived social support at T2 or T3, and a higher self-efficacy at T3 to predict higher life-satisfaction at T4 (Hypothesis 1) and less loneliness at T4 (Hypothesis 2). To further explore the potential conceptual overlap between general and source-specific social support, we conducted an additional regression analysis excluding the general support scale. This model, though not preregistered, was theoretically motivated and revealed that parental support remained a significant predictor of life satisfaction alongside self-efficacy. Full results are presented in the Additional Analyses section and Supplementary Tables S3, S4.

**Table 2:** Correlations of all predictors and the dependent variables life-satisfaction and loneliness.

Variable	1	2	3	4	5	6	7	8	9
1. Life-satisfaction									
2. Loneliness	−0.55** (*n* = 167)								
3. Religious belief	−0.03 (*n* = 67)	−0.09 (*n* = 67)							
4. General social support	0.48** (*n* = 149)	−0.54** (*n* = 150)	−0.10 (*n* = 61)						
5. Teacher support	0.28** (*n* = 126)	−0.23* (*n* = 127)	0.07 (*n* = 56)	0.28** (*n* = 127)					
6. Parental support	0.51** (*n* = 153)	−0.36** (*n* = 154)	0.05 (*n* = 62)	0.35** (*n* = 140)	0.22* (*n* = 120)				
7. Classroom support	0.29** (*n* = 152)	−0.28** (*n* = 153)	0.22 (*n* = 62)	0.23** (*n* = 139)	0.38** (*n* = 119)	0.26** (*n* = 154)			
8. Peer support	0.38** (*n* = 153)	−0.58** (*n* = 154)	0.17 (*n* = 62)	0.52** (*n* = 140)	0.30** (*n* = 120)	0.42** (*n* = 155)	0.45** (*n* = 154)		
9. Self-efficacy	0.52** (*n* = 153)	−0.32** (*n* = 154)	0.01 (*n* = 62)	0.25** (*n* = 140)	0.15 (*n* = 120)	0.49** (*n* = 155)	0.35** (*n* = 154)	0.39** (*n* = 155)	
10. Gender	−0.18* (*n* = 159)	0.16^(*)^ (*n* = 159)	−0.10 (*n* = 68)	0.02 (*n* = 144)	0.03 (*n* = 122)	−0.19* (*n* = 146)	−0.17* (*n* = 145)	−0.03 (*n* = 146)	−0.15 (*n* = 146)

^(*)^ indicates *p* < 0.10, * indicates *p* < 0.05, ** indicates *p* < 0.01.

**Table 3:** Multiple regression analysis of life-satisfaction.

Variable	Unstandardized coefficients		Standardized coefficients	*t*	*p*	*R* ^2^
	*b*	*SE_b_*	Beta (β)			
(Intercept)	0.11	0.67		0.16	0.875	
General social support	0.36	0.11	0.28	3.10	0.003**	
Teacher support	0.11	0.09	0.10	1.16	0.250	
Parental support	0.27	0.11	0.21	2.45	0.016*	
Classroom support	−0.19	0.13	−0.14	−1.43	0.156	
Peer support	0.12	0.19	0.06	0.63	0.529	
Self-efficacy	0.65	0.15	0.38	4.33	<0.001***	
Gender	−0.28	0.19	−0.11	−1.47	0.146	0.42***

*R*^2^ = adjusted *R*^2^. * indicates *p* < 0.05, ** indicates *p* < 0.01, *** indicates *p* < 0.001. *n* = 110.

**Table 4:** Multiple regression analysis of loneliness.

Variable	Unstandardized Coefficients		Standardized Coefficients	*t*	*p*	*R* ^2^
	*b*	*SE_b_*	Beta (β)			
(Intercept)	4.20	0.33		12.63	<0.001***	
General social support	−0.14	0.06	−0.22	−2.45	0.016*	
Teacher support	−0.00	0.05	−0.01	−0.08	0.937	
Parental support	−0.04	0.05	−0.07	−0.80	0.426	
Classroom support	−0.03	0.07	−0.04	−0.40	0.692	
Peer support	−0.40	0.09	−0.40	−4.21	<0.001***	
Self-efficacy	−0.08	0.07	−0.10	−1.11	0.271	
Gender	0.15	0.10	0.12	1.56	0.121	0.41***

*R*^2^ = adjusted *R*^2^. * indicates *p* < 0.05, *** indicates *p* < 0.001. *n* = 112.

General social support and self-efficacy emerged as significant predictors of life-satisfaction (*β* = 0.36, *p* = 0.003 and *β* = 0.65, *p* < 0.001 respectively). Adolescents who reported higher levels of self-efficacy and perceived social support during the study period also reported a greater life satisfaction at T4. Moreover, parental support showed significant predictive power on an adjusted alpha level (*β* =  0.27, *p* =  0.016). The initial main effect for gender disappeared in the multiple regression model. The model explained a significant proportion of variance in the post-lockdown life-satisfaction scores, *R*^2^ = 0.42, 95% CI [0.28,0.54], *p* < 0.001 and revealed a high power of 0.99 in the *post hoc* power analysis. Consequently, Hypothesis 1 could be partially confirmed.

With respect to loneliness, only peer support emerged as significant predictor of loneliness (*β* = −0.40, *p* < 0.001). Adolescents who had reported a better peer support at T3 were significantly less lonely at T4. General social support predicted loneliness as well, but the effect of *β* = −0.14, *p* = 0.016 was insignificant using our 1%-criterion. The model explained a significant proportion of variance in the T4 loneliness scores, *R*^2^ = 0.41, 95%, CI [0.27, 0.53], *p* < 0.001. Again, the post hoc power analysis revealed a high power of 0.99. Consequently, Hypothesis 2 could be partially confirmed.

Since the preliminary analyses had not revealed any significant correlations for Research Questions 3A and 3B (cf. Table 5) exploring whether a disposition to help predicts loneliness and life-satisfaction, we did not conduct any further analyses. In our adolescent sample, we could not identify any relations of prosociality or empathic concern at T4 with loneliness or life-satisfaction at T4.

**Table 5:** Correlations of exploratory predictors and the dependent variables life-satisfaction and loneliness.

Predictor	Outcome	
	Life-satisfaction	Loneliness
Perspective taking	−0.01 (*n* = 168)	−0.01 (*n* = 168)
Active-helping behavior	0.06 (*n* = 169)	−0.12 (*n* = 168)
Emotional-empathetic behavior	0.03 (*n* = 168)	−0.15 (*n* = 168)

^(*)^ indicates *p* < 0.10, * indicates *p* < 0.05, ** indicates *p* < 0.01.

### Additional analyses

As already mentioned in the *Participants* section, the final sample did not show significant differences in the variables of interest compared to participants who did not complete T4. Furthermore, during our analysis, we considered whether the general support scale encompassed effects of the various subscales, potentially leading to the loss of crucial information. As already mentioned, to address this conceptual overlap more explicitly, we conducted additional analyses, which are detailed in the supplement (cf. Supplementary Tables S3, S4). Analyses excluding the general social support scale revealed that, alongside self-efficacy (*β*  =  0.54, *p* <  0.001), parental support significantly predicted life-satisfaction (*β* =  0.35, *p* <  0.001). The model still explained a significant proportion of variance in the post-lockdown life-satisfaction scores, *R*^2^ = 0.38, 95% CI [0.26,0.49], *p* < 0.001. The result pattern of the new model explaining loneliness did not differ from the original model. Furthermore, potential interaction effects between the different predictors and gender were examined (cf. Supplementary Tables S5, S6) and discussed in the Supplementary material (cf. p. 10).

## Discussion

Building on evidence that the COVID-19 pandemic adversely affected adolescent wellbeing, the primary objective of this study was to examine whether adolescents’ wellbeing – assessed after the second lockdown with school-closings and at the end of the transition phase – could be predicted by different resources. Additionally, we explored whether a disposition to help, characterized by active helping behavior, emotional empathy, and perspective-taking, was associated with adolescents’ wellbeing at T4. Resources under investigation included religious belief, self-efficacy, and various forms of social support, all of which have been identified as protective factors during the pandemic (Agbaria and Abu-Mokh, 2022; Batmaz and Meral, 2022; Hussong et al., 2021; Panteli et al., 2021; Qi et al., 2020; Szkody et al., 2021). Adolescent wellbeing was operationalized through measures of life-satisfaction and (lack of) loneliness, both assessed at T4.

In line with Hypothesis 1, self-efficacy and general social support significantly predicted higher life-satisfaction at T4 (*p* < 0.01). Additionally, parental support at T3 emerged as a significant predictor of life-satisfaction when applying a standard significance criterion of *p* < 0.05. Contrary to expectations, religious belief, as well as teacher, peer, and classroom support, did not predict life-satisfaction. With respect to loneliness, consistent with Hypothesis 2, peer support emerged as a significant predictor of lower loneliness at T4 (*p* < 0.01), and general social support demonstrated predictive power at a standard significance criterion (*p* < 0.05). However, self-efficacy, teacher support, parental support, and classroom support did not show significant associations with loneliness. Thus, Hypotheses 1 and 2 were partially confirmed, as only some resources were found to be relevant predictors of life-satisfaction and loneliness. Notably, both regression models explained substantial proportions of the variance in the outcomes.

Regarding personal resources, perspective-taking, active helping behavior, and emotional-empathetic behavior did not predict life-satisfaction or loneliness, as these factors were not significantly correlated with either outcome.

### Adolescents’ life-satisfaction and loneliness after COVID-related lockdowns with school-closings

Prior research suggests that children, adolescents, and young adults worldwide were specifically affected by COVID-related lockdowns, experiencing heightened mental health challenges, reduced life satisfaction, and increased feelings of loneliness (Bu et al., 2020; Haikalis et al., 2022; Viner et al., 2022; Werling et al., 2022). Adolescents, in particular, faced significant disruptions as school-closings and changes to their social life, leisure activities, and daily routines dramatically altered their developmental environment. Given that adolescence is a critical period for the development of independence, with social interactions and activities outside the family playing a pivotal role, these restrictions posed unique burdens.

Nonetheless, studies across nations highlight competencies and conditions that may have protected adolescents and young adults from declines in life-satisfaction and increased feelings of loneliness. Among these, the role of self-efficacy in mitigating negative effects of lockdowns remains underexplored, particularly in adolescents. Yıldırım and Güler (2022) demonstrated that Turkish adults’ confidence in their ability to effectively protect themselves against COVID-19 positively predicted mental health. Furthermore, Alat et al. (2023) found that higher psychological capital and an internal locus of control were significantly associated with enhanced mental health in adults in India. Similarly, self-efficacy has been identified as a key personal resource for coping with pandemic-related stress among Palestinian adults and Jordanian nurses (Agbaria and Abu-Mokh, 2022; Shahrour and Dardas, 2020). The present study builds on these findings by investigating the impact of self-efficacy on adolescents’ life-satisfaction following the second COVID-related lockdown, which featured an extended period of school-closings and a prolonged transition phase characterized by heterogeneous regulations and regionally varying school reopening procedures across federal states and administrative districts. Previous research has established that self-efficacy demonstrates at least moderate temporal stability (Caprara et al., 2013; van Diemen et al., 2020). This stability suggests that self-efficacy may have persisted throughout the lockdown and contributed to adaptive coping strategies and a smoother transition back to normal routines. Our findings underscore the role of adolescents’ self-efficacy as a protective factor for life-satisfaction, independent of gender^4^. However, the relationship between self-efficacy and life-satisfaction may not be unidirectional. Adolescents with higher levels of life-satisfaction might also perceive themselves as more self-efficacious during the second lockdown with school-closings. This potential bidirectionality warrants further exploration to better understand the interplay between these constructs during crisis situations.

Our findings confirm and extend previous research by demonstrating that general social support significantly predicts adolescents’ life-satisfaction after the second COVID-related lockdown with school-closings and at the end of the transition phase. Consistent with studies by Li et al. (2021), Qi et al. (2020), and Szkody et al. (2021), we observed a positive association between social support and adolescents’ life-satisfaction. Notably, we also identified a relationship between parental support and life-satisfaction (*p* = 0.027). As highlighted in the additional analyses section, parental support emerged as a highly significant predictor of life-satisfaction when general social support was excluded from the regression model. This aligns with findings by El-Zoghby et al. (2020), who reported that family support increased more substantially than peer support during the pandemic. While face-to-face contact with peers was limited during lockdowns and school closings, many adolescents likely maintained their friendships through digital channels (Cauberghe et al., 2021), suggesting a shift in the form – rather than a breakdown – of peer interactions. Nevertheless, the reduced opportunities for in-person exchange may have diminished the emotional quality or perceived availability of peer support. As a result, family support likely gained heightened relevance for life satisfaction, even in domains where peer support typically plays a more dominant role.

Our findings further corroborate recent studies across diverse countries, societies, and age groups that identify social support as a predictor of loneliness during the pandemic (Bareket-Bojmel et al., 2021; Labrague et al., 2021). A notable contribution of our study lies in the differentiation of social support types, offering an important extension of existing literature. These distinctions are particularly valuable given the varying degrees to which different forms of social support were constrained during lockdowns and the subsequent transition phase. In contrast to life-satisfaction, our findings underscore the buffering role of peer support in mitigating loneliness among adolescents. This is especially important as social support from peers unavoidably declined during lockdowns and feelings of loneliness increased significantly (Rogers et al., 2021). Despite these challenges, our results emphasize the protective role of peer support and the importance of maintaining (digital) communication with friends. In line with Espinoza and Hernandez (2022), we observed that digital and verbal peer support acted as a resource against loneliness. While we did not assess the modality of peer interactions directly, it is likely that a substantial portion of social contact occurred digitally during lockdowns. Interestingly, prior research by Ellis et al. (2020) suggested that virtual interactions with friends during lockdowns were associated with both reduced loneliness and increased depression. In contrast, our findings indicate a stable, positive relationship between peer support and wellbeing, even under conditions of physical restriction. This may suggest that the quality of peer interactions – regardless of modality – is a decisive factor in their effectiveness as a protective resource.

Our findings highlight peer support – which likely occurred primarily through digital communication during lockdowns – as a protective resource against loneliness, while general social support and parental support emerged as more critical for adolescents’ life satisfaction. One key strength of the present study is its nuanced measurement of different dimensions of social support. First, the results suggest that various individuals or social groups contribute differently to distinct facets of adolescents’ wellbeing. Furthermore, our analysis suggests that when examining multiple aspects of social support, future studies should either avoid including a general social support variable or conduct supplementary analyses excluding this variable to reduce potential overlap or confounding effects. Second, the findings emphasize the importance of distinguishing between different aspects of wellbeing—such as loneliness and life-satisfaction—rather than treating wellbeing as a single, overarching construct.

Other resources, such as religious belief and teacher support, did not significantly predict adolescents’ loneliness or life-satisfaction in our study. The limited impact of religious belief may be attributed to the low proportion of religious participants in our sample. Prior research on the protective role of religious belief during the pandemic has predominantly involved traditionally Muslim-dominated populations, where faith tends to play a more central role in daily life (Tromp et al., 2022). In our study, only 40.11% of participants identified themselves as religious to any degree, which may have affected the potential effects of this resource. The abrupt shift from in-person to distance learning also posed substantial challenges for teachers (Marek et al., 2021), potentially limiting their ability to provide their usual level of support. Nonetheless, we found small to moderate correlations between teacher support and both life-satisfaction and loneliness. Adolescents with more supportive teachers appeared to benefit in these domains, suggesting that while teacher support is connected to adolescents` wellbeing, it is family and peer support that exert stronger effects on life-satisfaction and loneliness. However, it is important to note that some participants may have completed the surveys during school holidays, when regular contact with teachers was limited or absent. This temporal variation may have influenced how adolescents interpreted and rated teacher support, potentially affecting the strength and reliability of this predictor. At the same time, the items of the teacher support scale were phrased in a rather general manner, referring to the teacher who currently teaches the adolescent the most, rather than focusing on specific recent interactions. This may have enabled participants to provide meaningful responses even in the absence of recent or daily in-person contact.

Our study demonstrates that self-efficacy and various forms of social support served as protective factors against adolescents’ loneliness and as resources helping to sustain for maintaining life-satisfaction at the end of the second COVID-related lockdown with school-closings and the transition phase. These findings align with research suggesting that many adolescents demonstrated psychological resilience during the pandemic (Breaux et al., 2021; Krause al., 2021). While the present study focused on protective resources rather than risk trajectories, it may help explain why some adolescents maintained relatively stable levels of wellbeing despite the challenges of lockdowns and school disruptions. Furthermore, our findings point to actionable recommendations for preparing for future lockdowns or other significant life events. Specifically, fostering adolescents’ self-efficacy should be prioritized in both prevention and intervention programs. Moreover, structural measures should be implemented to strengthen adolescents’ social support networks, particularly for those from disadvantaged families, to enhance their resilience in the face of adversity.

Importantly, the associations observed in the present study should not be interpreted as uniquely pandemic-specific. A substantial body of research has demonstrated robust links between social support, self-efficacy, and adolescent wellbeing across diverse contexts (Chu et al., 2010; Compas et al., 2017). In particular, the negative association between perceived social support and loneliness, as well as the central role of peer relationships for adolescents’ social wellbeing represent developmentally normative patterns (Nickerson and Nagle, 2005). In this light, our findings suggest that core developmental mechanisms continued to operate even during extended phases of societal restriction. The stability of these associations under conditions of disrupted schooling and reduced in-person interaction may therefore be theoretically meaningful. At the same time, the comparatively pronounced role of parental support for life-satisfaction may reflect context-sensitive shifts in the relative salience of support sources when peer contact was structurally constrained. This interpretation is consistent with prospective pandemic research highlighting the continued importance of established protective factors (Magson et al., 2021).

### Life-satisfaction, loneliness, and disposition to help

Our findings do not relate life-satisfaction to loneliness and a disposition to help (Research Questions 3A and 3B). While previous studies suggested that both constructs are associated with greater compliance with COVID-19 protective measures (Jordan et al., 2021; Qu et al., 2022), our results indicate that a disposition to help, as a personal resource, did not predict life-satisfaction or loneliness. In contrast, resources such as staying connected with peers, family support, and self-efficacy were found to predict life-satisfaction and buffer against loneliness. Prior research has shown a decline in adolescents’ empathic concern during the initial weeks of the first lockdown (Van de Groep et al., 2020). Our findings may support the hypothesis that during periods of heightened self-focus, empathic and prosocial tendencies do not contribute significantly to wellbeing, whereas self-focused resources like self-efficacy and social support play a more critical role.

### Limitations, implications, and future directions

This study has important limitations. First, the reduction of the sample size to *N* = 171 in the main analyses poses challenges typical of small sample studies (Button et al., 2013). However, the observed high effect sizes and sufficient statistical power lend support to the reliability of our findings. Second, the predominantly Christian composition of the sample might have influenced the observed levels of religiosity, potentially limiting generalizability. Third, due to variations in COVID-19 regulations across German federal states and differing assessment timelines, there is uncertainty regarding the lockdown status of individual participants. The dynamic interplay between lockdown and transition phases, with varying levels of in-person schooling, may have influenced participants’ perceptions of social support. In particular, adolescents who completed a survey during summer vacations (measurement distance was 8 weeks, summer vacations were 6 weeks, thus only one assessment may have been during vacations) may have experienced substantially different patterns of peer and teacher contact compared to those surveyed during school terms. Fourth, the study does not provide insights into the long-term effects of the second COVID-19 lockdown or changes in wellbeing over time, as loneliness and life-satisfaction were each assessed only once, post-lockdown and at the end of the transition phase. Fifth, our design does not permit causal conclusions regarding the impact of the second lockdown on the reported outcomes, as changes in the dependent variables were not captured longitudinally and no pre-pandemic baseline data were available. Sixth, although we controlled for gender effects in our analyses, the overrepresentation of female participants in our sample may have affected the findings. Seventh, another limitation of the current study is the reduction in sample size due to attrition and the use of listwise deletion for participants with missing outcome data at T4. This analytic decision followed our preregistered plan and was necessary, as outcome variables were assessed only at this final timepoint. While alternative approaches such as imputation or FIML are often discussed in the context of missing data, their application is limited in designs where outcomes are not measured repeatedly. In such cases, listwise deletion remains an appropriate and commonly used method in psychological research, particularly when missingness is low in the key analytic variables and outcome data are not available for reliable estimation (Graham, 2009). Eighth, while we interpret our findings in the context of COVID-19-related lockdowns and school closings, we acknowledge that adolescents’ wellbeing during the pandemic may have been influenced by a broader range of factors not captured in this study. These include fear of infection, illness or loss within the family, economic hardship, general uncertainty about the future, disruptions to daily routines such as the cancellation of hobbies (e.g., team sports or group music-making), strained family dynamics due to remote work and schooling, and the emotional burden of being separated from vulnerable family members such as grandparents. As our study did not assess these aspects directly, we cannot definitively attribute changes in wellbeing solely to school closings or lockdown measures. Future research should incorporate a wider range of pandemic-related stressors to more fully disentangle their relative contributions. Ninth, the interpretation of teacher support must be approached with caution, as some participants may have completed surveys during school holidays. This temporal overlap may have affected both the availability and relevance of teacher support, possibly limiting the interpretability of results related to this predictor. Lastly, the reliance on self-reported measures may have caused common method bias, potentially influencing data quality. Nonetheless, the study design incorporated measures to reduce such bias, including the use of separate assessment times for predictors and outcomes, clear and motivating instructions, and reverse-coded items (Jordan and Troth, 2020).

Despite these limitations, this study offers several important contributions. By focusing on the second COVID-19 lockdown and the subsequent transition phase, it addresses a less frequently examined period of the pandemic and provides new insights into adolescents’ wellbeing during a time of ongoing disruption and uncertainty. Our results underscore the critical role of self-efficacy and diverse types of social support as important protective resources for adolescents during the later phases of the COVID-19 pandemic, including periods of physical and social restriction. These findings lay the groundwork for the development of targeted interventions aimed at strengthening adolescents’ resilience and enhancing their access to effective coping resources in future crises or major life disruptions. Future research could also examine how cultural and religious diversity may shape adolescents’ access to and use of such coping resources, and how these factors influence psychological adjustment and wellbeing in times of crisis. Given the relatively low levels of religiosity in our sample, it is possible that different patterns might emerge in more religiously diverse or devout populations. Additionally, the concept of coronaphobia – pandemic-specific fear driven by uncertainty, health concerns, and media exposure (Arora et al., 2020) – may offer a useful lens for understanding individual differences in adolescents’ stress responses during such disruptive periods. Finally, our findings have two conceptual advances: First, different forms of social support and personal resources appear to be differentially associated with specific facets of adolescents’ wellbeing, emphasizing the need for more tailored, domain-specific interventions. Second, the results highlight the importance of treating wellbeing as a multidimensional construct rather than a unitary outcome in future research and practice.

## Data Availability

The datasets presented in this study can be found in online repositories. The names of the repository/repositories and accession number(s) can be found below: https://osf.io/vzpqk.

## Footnotes

1
https://osf.io/vzpqk

2 A list including all items can be requested from the authors. For copyright reasons we may not reprint the items used.

3 The R-Scripts for all analyses used in this study can be found at https://osf.io/c8rkp/?view_only=89a721e5e01042c89abce76b7ee2a994

4 A discussion regarding a potential interaction between self-efficacy and gender is available in the supplement since we did not preregister the calculation of potential interaction effects.
