# Supplementary material for: Adolescent wellbeing after COVID-related school-closings
Source: Front Psychol. 2026 Mar 27;17:1591897. doi: 10.3389/fpsyg.2026.1591897 (PMC13065659; doi:10.3389/fpsyg.2026.1591897)
Supplement: Supplementary file 1 [file Data_Sheet_1.pdf]

Supplemental Material

Adolescent Well-being After COVID-related School-closings – a resourced-based view

Content **Page**

Deviations from Preregistration

Table S1 *Deviations from the Preregistration* 2

Preliminary Analyses

Confirmatory Factor Analyses for *Disposition to Help* 4

Main Analyses

Table S2 *Final Sample Sizes* 5

Additional Analyses

Table S3 *Multiple Regression Analysis of Life-satisfaction Without General and Teacher support* 6

Table S4 *Multiple Regression Analysis of Loneliness Without General and Teacher support* 7

Table S5 *Multiple Regression Analysis of Life-satisfaction With Gender Interaction* 8

Table S6 *Multiple Regression Analysis of Loneliness With Gender Interaction* 9

Discussion of a Potential Self-efficacy With Gender Interaction …10

Reviewer Suggestions …11

Table S7 *Responses During the Nationwide Summer Vacation Dates vs. Total Sample* 13

Table S8 *Descriptive Statistics for T4 Outcomes: Summer Vacation Dates vs. all Other Dates* 14

Figures

Figure S1 *Model 1 With Disposition to Help With One Factor* 15

Figure S2 *Model 2 With Disposition to Help With Three Different Factors* 16

References 17

Table S1

Deviations from the Preregistration [ADD LINK UPON ACCEPTANCE]

Preregistration	Deviation	Reason for deviation
"Preliminary Question: Are adolescent's level of life-satisfaction and loneliness (2 of 3 dependent variables in this study) different to life-satisfaction and loneliness of a same-age sample collected prior to and during the first COVID-related lockdown?"	We removed the same-age sample from the main analyses.	Given that the comparison of two different samples has proven to be conceptually inappropriate, we have decided not to report any results in this regard.
We had preregistered competence-gain as one 7-item scale. Thus, we planned all analyses with competence-gain as one scale.	We removed the competence-gain scale from all analyses.	We conducted factory analyses for the competence-gain scale. We first conducted EFA to explore a possible factor structure. We subsequently tested the fit of the resulting two-factor structure of the scale in a CFA. The poor fit of the different models let us remove the self-developed scale from all analyses.
We did not preregister the comparison of finishers and non-finisher of the main sample.	We added t-tests to compare participants without the critical scale scores at T4 with those of the final sample.	We conducted t-tests to ensure more data quality and to be able to interpret the data more correctly.
We did not preregister the analyses without general support and teacher support.	We added regression models without general support and teacher support.	We conducted additional regression models to be able to interpret the data more correctly. During analysis, we considered whether the general support scale encompasses effects of the various subscales, potentially leading to the loss of crucial information.

We did not preregister the analyses of potential gender interactions.	We added regression models with interactions between gender and the predictors.	We conducted additional regression models to be able to interpret the data more precisely. Since we found main effects for gender in the DV, we tested for interaction effects with the different predictors.

Confirmatory Factor Analyses for *Disposition to Help*

Using the estimator WLSMV in the R-package *lavaan* (Rosseel, 2012), we conducted a confirmatory factor analysis to test whether the two facets of prosociality and perspective taking with 3 items each loaded on one factor, thus representing disposition to help as one construct. We used TLI ≥ .95, RMSEA ≤ .08, and SRMR ≤ .10 as requirements for satisfying model fit (Schermelleh-Engel et al., 2003). The confirmatory factor model with all 9 items loading on one factor did not yield satisfying model fit (χ^2_{27} = 95.32, TLI = .643, RMSEA = .123 SRMR = .087). These results indicate that there is no unidimensional disposition to help. We had preregistered to treat the three scales separately as different aspects favoring a disposition to help. We thus checked this structure in a second confirmatory factor analysis with active-helping, empathic concern, and perspective taking as three separate factors with three items each. The three-factor model yielded good fit (χ^2_{24} = 38.00, TLI = .92, RMSEA = .06, SRMR = .046). The TLI value was lower than the criterion we had preregistered as acceptable for treating the 9 items as a unidimensional scale (TLI ≥ .95), but the very good RMSEA and SRMR values indicate that it was statistically correct to treat active-helping behavior, emotional-empathetic behavior, and perspective taking as separate scales. The path diagrams for both models are displayed in Figures S1 and S2.

Table S2

Final Sample Sizes

Measure	Time of Assessment	Participants
Life-satisfaction	T4	*N* = 169
Loneliness	T4	*N* = 169
Religious belief	T1	*N* = 68
General social support	T2	*N* = 151
Teacher support	T2	*N* = 128
Parental support	T3	*N* = 155
Peer support	T3	*N* = 155
Classroom support	T3	*N* = 154
Self-efficacy	T3	*N* = 155
Perspective-taking	T4	*N* = 170
Active-helping behavior	T4	*N* = 170
Emotional-empathetic behavior	T4	*N* = 170

Note. The scale teacher support was represented to pupils only. The SBI had not been displayed to all participants. There was a filter question asking participants to rate their religiosity on a scale from 1 (not at all) to 5 (very much). Only those who had selected 3 or higher were displayed the SBI and could have obtained a scale mean when answering all items.

Table S3

Multiple Regression Analysis of Life-satisfaction Without General and Teacher support

Variable	Unstandardized Coefficients		Standardized Coefficients	t	p	R^2
	b	SE_b	Beta (β)			
(Intercept)	1.10	.55			<.05*	
Parental support	.35	.10	.29	3.66	<.001***	
Classroom support	-.01	.08	-.01	-.16	.875	
Peer support	.28	.14	.15	1.88	.062	
Self-efficacy	.54	.12	.34	4.36	<.001***	
Gender	-.25	.16	-.10	-1.53	.128	
						.38***

Note. R^2 = adjusted R^2. * indicates p < .05, ** indicates p < .01, *** indicates p < .001. n = 143

Table S4

Multiple Regression Analysis of Loneliness Without General and Teacher support

Variable	Unstandardized Coefficients		Standardized Coefficients	t	p	R^2
	b	SE_b	Beta (β)			
(Intercept)	4.00	.28			<.001***	
Parental support	-.07	.05	-.12	-1.50	.137	
Classroom support	.01	.04	.02	.22	.824	
Peer support	-.52	.08	-.53	-6.86	<.001***	
Self-efficacy	-.09	.06	-.10	-1.34	.182	
Gender	.13	.08	.10	1.55	.123	
						.40***

Note. R^2 = adjusted R^2. * indicates p < .05, ** indicates p < .01, *** indicates p < .001. n = 144

Table S5

Multiple Regression Analysis of Life-satisfaction With Gender Interaction

Variable	Unstandardized Coefficients		Standardized Coefficients	*t*	*p*	R^2
	b	SE_b	Beta (β)			
(Intercept)	-.52	2.68			.847	
Parental support	1.29	.69	1.05	1.86	.066	
Classroom support	.42	.55	.38	.76	.450	
Peer support	-.72	.87	-.39	-.83	.409	
Self-efficacy	.58	.68	.37	.86	.390	
Gender	.61	1.40	.25	.43	.666	
Parental support x Gender	-.48	.36	-.92	-1.34	.181	
Classroom support x Gender	-.23	.29	-.47	-.81	.418	
Peer support x Gender	.53	.46	.87	1.17	.245	
Self-efficacy x Gender	-.04	.36	-.07	-.12	.905	.38

Note. R^2 = adjusted R^2. * indicates p < .05, ** indicates p < .01, *** indicates p < .001. *n* = 143

Table S6

Multiple Regression Analysis of Loneliness With Gender Interaction

Variable	Unstandardized Coefficients		Standardized Coefficients	*t*	*p*	R^2
	b	SE_b	Beta (β)			
(Intercept)	4.71***	1.36			<.001***	
Parental support	-.56	.36	-.88	-1.56	.120	
Classroom support	-.26	.25	-.46	-1.06	.293	
Peer support	-.02	.45	-.02	-.05	.961	
Self-efficacy	.01	.31	.02	.04	.968	
Gender	-.24	.71	-.19	-.34	.735	
Parental support x Gender	.25	.19	.92	1.53	.178	
Classroom support x Gender	.15	.13	.57	1.12	.267	
Peer support x Gender	-.26	.23	-.83	-1.13	.262	
Self-efficacy x Gender	-.04	.17	-.13	-.26	.796	.39***

Note. R^2 = adjusted R^2. * indicates p < .05, ** indicates p < .01, *** indicates p < .001. *n* = 143

Discussion of Potential Predictor with Gender Interactions

Although there were no main effects of gender in the multiple regression analyses for the dependent variables life-satisfaction and loneliness, the findings in Tables S7 and S8 suggest gender might have a relevant influence on the relationship between predictors and outcomes. When including interaction terms between the predictors and gender into our model, the main effects reduced in both models. This indicates that gender might moderate the relationship between predictors and outcomes. *Preliminary analyses* had revealed gender-specific trends: male gender was associated with higher life-satisfaction, while female gender predicted higher loneliness. These findings align with prior research findings regarding higher life-satisfaction among boys during adolescence (Chen et al., 2020). However, the literature on gender differences in loneliness during adolescence remains inconsistent with contradictory findings (Maes et al., 2019). Our observation of increased loneliness among girls corroborates findings from the COVID-19 pandemic, which highlighted increased loneliness in girls and women (Bu et al., 2020; Lee et al., 2020; Lepinteur et al., 2022).

Our findings suggest that gender may influence the relationship between predictors and outcomes, indicating that adolescents' resources might affect life-satisfaction and loneliness differently based on gender. Such findings highlight the importance of considering gender as an influencing variable in future research on adolescent well-being. While gender did not emerge as a significant main effect in our regression models, its potential influence merits further exploration, especially in studies investigating the role of resources in shaping life-satisfaction and loneliness during adolescence or crisis.

Reviewer Suggestions

To address a reviewer's suggestion, we conducted an additional date-specific sensitivity analysis to identify potential biases related to summer vacation timing. Specifically, we identified the only dates in 2021 during which all German federal states were simultaneously on summer vacation (31 July - 01 August 2021). This window represents the only period in which responses can be unequivocally classified as occurring during school holidays nationwide. Table S7 presents the number of responses within this nationwide vacation dates across measurement waves. No responses fell into this time window at T1 and T3. At T2, 2 responses were recorded, and at T4, 10 responses occurred during this period. Given the small cell sizes – particularly for T2 – robust statistical comparisons are not feasible for most waves. For T4, where the number of responses within the nationwide vacation dates was comparatively larger (n = 10), we additionally report descriptive statistics for life-satisfaction and loneliness for responses collected during this vacation dates compared to all other T4 responses (Table S8). Because the comparison group necessarily includes a mixture of school periods, transition phases, and potentially additional holiday periods (which cannot be precisely identified due to the absence of regional location data), these comparisons are purely descriptive and do not constitute a definitive test of vacation versus non-vacation effects. Overall, this additional descriptive analysis serves to transparently document the temporal distribution of responses and to contextualize potential timing effects, rather than to draw inferential conclusions about school holiday impacts. Importantly, the descriptive means for life-satisfaction and loneliness did not differ substantially between responses collected during the nationwide vacation dates and those collected at other T4 time points, suggesting that there is no clear indication of pronounced vacation-related distortions in these outcomes within the limits of the available data.

In addition to examining potential vacation effects, we also revisited the reviewer's suggestion to exclude teacher support and classroom support from the regression models, given that some assessments may have occurred during school holidays. In our preregistered main analyses, teacher support was included as a theoretically relevant school-related resource. However,

during the analytic process, we already considered the possibility that the temporal heterogeneity of data collection (i.e., potential overlap with school holidays) might attenuate or distort effects related to teacher support. For this reason, teacher support was excluded from the alternative regression model reported in the Additional Analyses section. Following the reviewer's suggestion, we additionally estimated a revised regression model excluding both teacher support and classroom support to further minimize potential timing-related interpretational constraints. This model retains self-efficacy, parental support, and peer support as predictors.

When teacher and classroom support were excluded from the regression model predicting life-satisfaction at T4, self-efficacy remained a significant positive predictor (β = .54, p < .001). Parental support also remained significant (β = .35, p < .001), while peer support showed non-significant effects (β = .27, p = .05). The overall model explained a substantial proportion of variance, R^2 = .41, 95% CI [.27,.49], p < .01. Similarly, for loneliness at T4, self-efficacy (β = -.09, p = .17) and parental support (β = -.07, p = .15) remained non-significant predictors in the regression model while peer support was a significant predictor (β = -.51, p < .001). The model accounted for R^2 = .41, 95% CI [.28,.50], p < .001 of the variance in loneliness. Importantly, the pattern and magnitude of effects were highly comparable to the previously reported models (R^2 = .42, 95% CI [.28,.54], p < .001 for life-satisfaction; R^2 = .41, 95%, CI [.27, .53], p < .001 for loneliness), suggesting that the main conclusions of the study do not depend on the inclusion of school-related support variables. These additional analyses therefore reinforce the robustness of the reported associations.

Table S7

Responses During the Nationwide Summer Vacation Dates vs. Total Sample

Wave	*n* (vacation)	*N* (total)
T1	0	161
T2	2	152
T3	0	155
T4	10	171

Note. Counts refer to the only dates in 2021 (31 July–01 August) during which schools in all German federal states were on summer vacation.

Table S8

Descriptive Statistics for T4 Outcomes: Summer Vacation Dates vs. all Other Dates

Outcome	Vacation (*n*)	Vacation (*M*)	Vacation (*SD*)	Other (*n*)	Other (*M*)	Other (*SD*)
Life-satisfaction	10	3.89	.87	159	4.16	.98
Loneliness	10	2.19	.61	159	2.16	.51

Note. The summer vacation dates refers to 30 July–01 August 2021 (nationwide overlap across German federal states).

Figure S1

Model 1 With Disposition to Help With one Factor

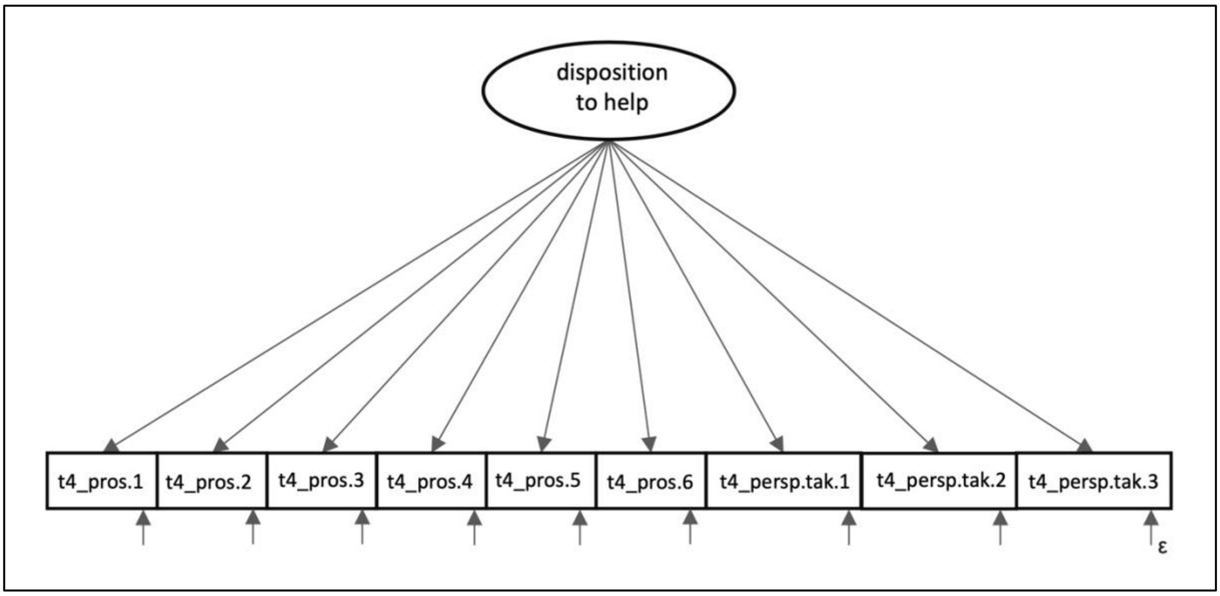

Note. The items are taken from the following subscales: active-helping behavior: t4_pros.1, t4_pros.2, t4_pros.3; emotional-empathetic behavior: t4_pros.4, t4_pros.5, t4_pros.6; perspective taking: t4_persp.tak.1, t4_persp.tak.2, t4_persp.tak.3

Figure S2

Model 2 With Disposition to Help With Three Different Factors

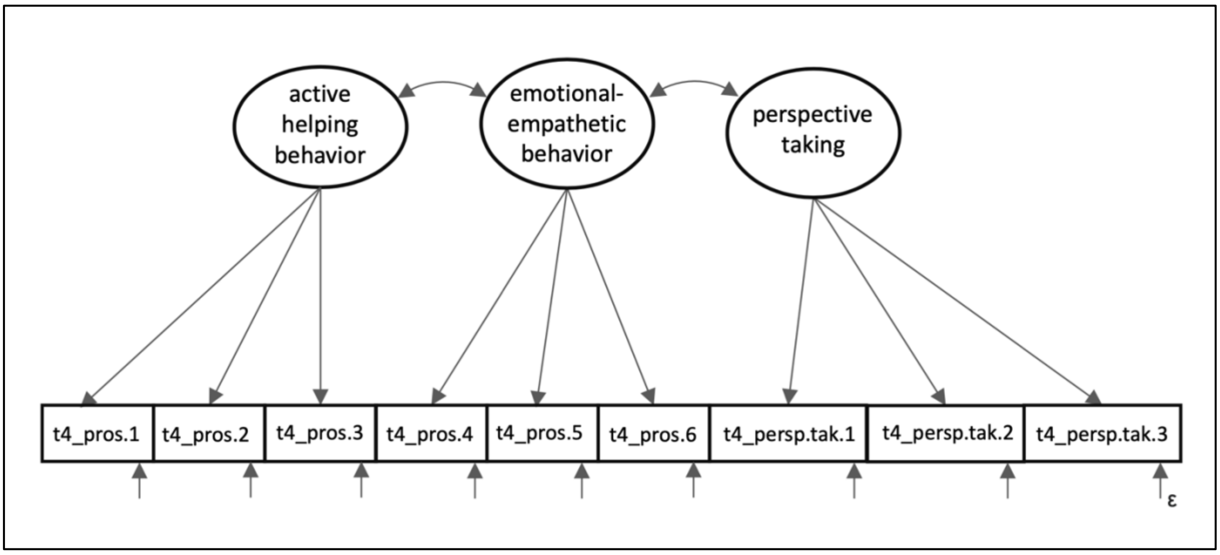

Note. The items are taken from the following subscales: active-helping behavior: t4_pros.1, t4_pros.2, t4_pros.3; emotional-empathetic behavior: t4_pros.4, t4_pros.5, t4_pros.6; perspective taking: t4_persp.tak.1, t4_persp.tak.2, t4_persp.ta

References

Bu, F., Steptoe, A., & Fancourt, D. (2020). Who is lonely in lockdown? Cross-cohort analyses of predictors of loneliness before and during the COVID-19 pandemic. *Public Health*, *186*, 31–34. https://doi.org/10.1016/j.puhe.2020.06.036

Chen, X., Cai, Z., He, J., & Fan, X. (2020). Gender Differences in Life Satisfaction Among Children and Adolescents: A Meta-analysis. *Journal of Happiness Studies*, *21*(6), 2279–2307. https://doi.org/10.1007/s10902-019-00169-9

Lee, C. M., Cadigan, J. M., & Rhew, I. C. (2020). Increases in loneliness among young adults during the COVID-19 pandemic and association with increases in mental health problems. *Journal of Adolescent Health*, *67*(5), 714–717. https://doi.org/10.1016/j.jadohealth.2020.08.009

Lepinteur, A., Clark, A. E., Ferrer-i-Carbonell, A., Piper, A., Schröder, C., & D'Ambrosio, C. (2022). Gender, loneliness and happiness during COVID-19. *Journal of Behavioral and Experimental Economics*, *101*, 101952. https://doi.org/10.1016/j.socec.2022.101952

Maes, M., Qualter, P., Vanhalst, J., Van den Noortgate, W., & Goossens, L. (2019). Gender Differences in Loneliness across the Lifespan: A Meta–Analysis. *European Journal of Personality*, *33*(6), 642–654. https://doi.org/10.1002/per.2220

Rosseel, Y. (2012). lavaan: An R Package for Structural Equation Modeling. *Journal of Statistical Software*, *48*(2), 1–36. https://doi.org/10.18637/jss.v048.i02

Schermelleh-Engel, K., Moosbrugger, H., & Müller, H. (2003). Evaluating the fit of structural equation models: Tests of significance and descriptive goodness-of-fit measures. *Methods of Psychological Research Online*, *8*(2), 23–74.
